# Spontaneous Blinks Activate the Precuneus: Characterizing Blink-Related Oscillations Using Magnetoencephalography

**DOI:** 10.3389/fnhum.2017.00489

**Published:** 2017-10-11

**Authors:** Careesa C. Liu, Sujoy Ghosh Hajra, Teresa P. L. Cheung, Xiaowei Song, Ryan C. N. D'Arcy

**Affiliations:** ^1^School of Engineering Science, Simon Fraser University, Burnaby, BC, Canada; ^2^Health Science and Innovation, Surrey Memorial Hospital, Fraser Health Authority, Surrey, BC, Canada

**Keywords:** blink, blink-related oscillations, precuneus, environmental monitoring, awareness, magnetoencephalography

## Abstract

Spontaneous blinking occurs 15–20 times per minute. Although blinking has often been associated with its physiological role of corneal lubrication, there is now increasing behavioral evidence suggesting that blinks are also modulated by cognitive processes such as attention and information processing. Recent low-density electroencephalography (EEG) studies have reported so-called blink-related oscillations (BROs) associated with spontaneous blinking at rest. Delta-band (0.5–4 Hz) BROs are thought to originate from the precuneus region involved in environmental monitoring and awareness, with potential clinical utility in evaluation of disorders of consciousness. However, the neural mechanisms of BROs have not been elucidated. Using magnetoencephalography (MEG), we characterized delta-band BROs in 36 healthy individuals while controlling for background brain activity. Results showed that, compared to pre-blink baseline, delta-band BROs resulted in increased global field power (*p* < 0.001) and time-frequency spectral power (*p* < 0.05) at the sensor level, peaking at ~250 ms post-blink maximum. Source localization showed that spontaneous blinks activated the bilateral precuneus (*p* < 0.05 FWE), and source activity within the precuneus was also consistent with sensor-space results. Crucially, these effects were only observed in the blink condition and were absent in the control condition, demonstrating that results were due to spontaneous blinks rather than as part of the inherent brain activity. The current study represents the first MEG examination of BROs. Our findings suggest that spontaneous blinks activate the precuneus regions consistent with environmental monitoring and awareness, and provide important neuroimaging support for the cognitive role of spontaneous blinks.

## Introduction

Spontaneous eye blinks occur ~15–20 times per minute (Tsubota et al., 1996). While blinks have often been associated with their physiological role of corneal lubrication, there is now increasing behavioral evidence suggesting that blinks are also modulated by cognitive processes such as attention and information processing (Veltman and Gaillard, 1998; Viggiano and Mecacci, 2000; Oh et al., 2012). Spontaneous blinks tend to occur at moments when attentional demand is low, such as punctuation marks while reading (Hall, 1945; Orchard and Stern, 1991), pauses by the speaker while listening to speech (Nakano and Kitazawa, 2010), and at scenes containing less relevant information while watching videos (Nakano et al., 2009). Additionally, when compared to rest, tasks involving continued visual attention (e.g., reading and passive information viewing) lead to reduction in spontaneous blink rate, while those involving active information processing and memory (e.g., conversation and silent information rehearsal) lead to increased blink rate (Dejong and Merckelbach, 1990; Bentivoglio et al., 1997). Nonetheless, the endogenous neural mechanisms underlying cognitive aspects of spontaneous blinking have not been elucidated.

The current understanding of blink-related brain activity often centers on the blink motor command and the phenomenon of blink suppression, in which visual sensitivity is momentarily reduced over the duration of the blink (Volkmann, 1986; Bristow et al., 2005b; Berman et al., 2012). This phenomenon is believed to help mitigate the loss of sensory input due to blinking, thereby accounting for the behavioral observation that the frequent visual interruptions due to blinking are rarely noticed in the subjective experience (Volkmann, 1986). The physiological process of eye blinking occurs through contraction of the orbicularis oculi muscle and relaxation of the levator palpebrae superioris muscle in the face, producing a rapid closing and reopening of the eyelids that typically lasts ~150–300 ms in duration (Riggs et al., 1981; Manning et al., 1983). Functional MRI (fMRI) studies of voluntary blinking have observed brain activations across the occipital, posterior parietal, as well as prefrontal oculomotor regions including the frontal eye field and supplementary eye field (Bodis-Wollner et al., 1999; Tsubota et al., 1999; Kato and Miyauchi, 2003; Bristow et al., 2005b). On the other hand, spontaneous blinking has been shown to activate primarily the prefrontal oculomotor regions and the occipital cortex (Yoon et al., 2005; Berman et al., 2012; Hupe et al., 2012). Other studies using magnetocephalography (MEG) (Bardouille et al., 2006) and electroencephalography (EEG) (Heuser-Link et al., 1992) have reported blink-related activity in the frontally located eye regions—primarily attributed to muscle contractions and eye movement—as well as the occipital cortex.

In addition to its role in visual suppression, spontaneous blinking has also recently been proposed to engage certain areas of the default mode network (DMN) (Nakano et al., 2013), a network of brain regions exhibiting elevated activity in the absence of goal-directed tasks (Raichle et al., 2001). The DMN is involved in internally directed processes (Buckner et al., 2008), and is activated in both eyes-closed rest and eyes-open fixation states (Raichle et al., 2001). In the context of spontaneous blinking, fMRI research has demonstrated that blinks while viewing videos increased activity in regions of the DMN (including precuneus, posterior cingulate cortex, and angular gyrus), while simultaneously deactivating areas of the brain involved in top-down control of goal-directed attention (Nakano et al., 2013). The authors proposed that spontaneous blinks were involved in disengagement of attention during cognitive behaviors (Nakano et al., 2013).

Besides these fMRI findings, other evidence has also emerged from both MEG and EEG suggesting the blink-related activation of the precuneus region. Work using MEG demonstrated activation of the medial parieto-occipital sulcus region following voluntary blinks (Hari et al., 1994). In addition, EEG studies have reported blink-related oscillations (BROs) as electrophysiological phenomena derived from EEG data time-locked to blinks, in which trial-averaged delta band (0.5–4 Hz) signals (following removal of ocular artifact) exhibited a positive deflection occurring ~300 ms post-blink (Bonfiglio et al., 2009). The authors hypothesized that these delta-band BROs may be similar to the P300 event-related potential corresponding to attention orienting mechanisms in the brain (Polich, 2007; Bonfiglio et al., 2009). Delta BROs have also been suggested to originate from the posterior cingulate cortex and precuneus regions (Bonfiglio et al., 2013). The precuneus is a central hub within the DMN (Fransson and Marrelec, 2008; Hagmann et al., 2008), and is involved in visuo-spatial processing and awareness (Cavanna and Trimble, 2006). Moreover, further studies demonstrated that BRO spectral power differentiated between healthy control individuals and brain-injured patients with disorders of consciousness (DOC) (Bonfiglio et al., 2013, 2014). Given the importance of the DMN—and particularly the precuneus—in assessment of patients with DOC (Boly et al., 2009; Crone et al., 2011; Laureys and Schiff, 2012), these findings create an intriguing possibility that BROs may be utilized as a potential indicator of brain functional status in DOC evaluations.

Although delta BROs have been applied to small samples of brain-injured patients with promising results (Bonfiglio et al., 2013, 2014), their neurocognitive mechanisms are not well-understood. Previous BRO studies have been based on low-density EEG systems, which have significant limitations in spatial resolution due largely to the electrical field distortions that arise in volume conduction (Nunez et al., 1994). In contrast, magnetoencephalography (MEG) measures the magnetic fields generated by electrical current flow within neuronal assemblies, and magnetic field propagation is not affected by differences in tissue conductivities in the head (Marinova and Mateev, 2010). Combined with high sensor density and advanced source reconstruction algorithms, this advantage helps give MEG its superior spatial resolution compared to EEG (Brookes et al., 2011).

We conducted the first MEG investigation of BROs to examine their neurocognitive mechanisms in a large sample of healthy individuals, with particular emphasis on the delta band. We hypothesized that the time course of delta BRO activity would exhibit peak power within the 200–400 ms window post-blink, with localization to the precuneus regions. We further expected that these sensor-level characteristics would also be reflected in the source-level activity within the precuneus regions. Our findings confirm these hypotheses, and demonstrate that spontaneous blinking during resting fixation strongly activates the bilateral precuneus regions known to be associated with environmental monitoring and awareness (Cavanna and Trimble, 2006). Using a novel analytical approach to control for the timing of blink-related relative to background brain activity, we show that the observed effects were specifically due to spontaneous blink instances rather than as part of inherent brain activations during resting fixation. These results help provide further neuroimaging support for the potential cognitive importance of blink-related brain activity.

## Materials and methods

### Participants

Forty-one healthy control adults (age 23.8 ± 3.9, 20 female) participated in this study, all with normal or corrected-to-normal vision. This research was approved by the Research Ethics Boards of Simon Fraser University (Protocol 2014s0177) and Fraser Health Authority (Protocol 2014-076), and written informed consent was obtained from each participant prior to data acquisition.

### Data acquisition

During each collection, participants were instructed to rest comfortably in the supine position, and remain awake with their eyes fixated on a centrally presented cross (white on black background). Participants were not informed of the purpose of the study in order to acquire natural spontaneous blink responses. Data acquisition took place in a well-lit, magnetically shielded room. MEG data were collected using a whole-head CTF system (MEG International Services Ltd, Canada) with 151 axial gradiometers (5 cm baseline). Synthetic 3rd-order gradient noise cancelation was also employed. Data were sampled at 1,200 Hz frequency, and each run lasted 10 minutes. Head position was continuously recorded using energizing coils positioned at three fiducial points (nasion, left, and right periauricular). Prior to data acquisition, the participant's head shape was measured using a 3D digitizer (Polhemus, USA), with a minimum of 500 points acquired over the entire head. To enable tracking of blinks and eye movements, electrooculogram (EOG) was recorded from electrodes positioned near the left eye on the supra-orbital ridge (vertical EOG or vEOG) and outer canthus (horizontal EOG). Electrode impedances were kept below 5 kΩ. The EOG channel data were monitored online to ensure participants remained awake with eyes open throughout the experiment.

### Blink detection and evaluation

MEG and EOG data were visually inspected, and artifactual channels were removed. Data were then notch-filtered to remove 60 Hz power line noise and high frequency signal due to the head position indicator coils. Data from 5 of the 41 participants were removed from subsequent analyses due to poor quality. EOG data were band-pass filtered to 0.1–30 Hz, and blink events were automatically identified using a template matching procedure based on data from the vEOG channel (Bonfiglio et al., 2013). In particular, one blink template best representing a stereotypical blink was first manually identified for each individual, and the entire vEOG data series were convolved with the blink template. Amplitude thresholding was applied to the convolution signal to select potential blink instances, followed by temporal thresholding to exclude blink instances less than 3 s away from adjacent blinks in order to avoid contamination from activity due to other blinks. All final blink events were visually inspected to ensure artifact-free data.

**Figure 1:**
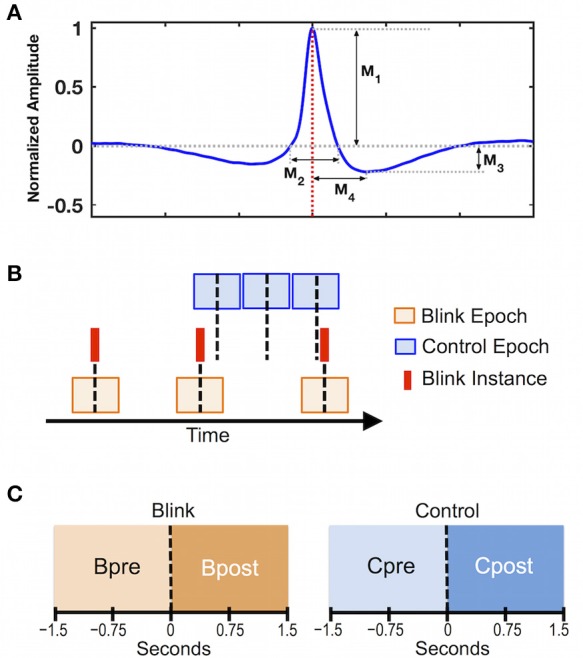
Data preprocessing steps. **(A)** Grand average of 3 s blink epochs in vEOG recording showing the morphological features used in behavioral evaluation. Features describe the height and width of the positive and negative portions of the blink trace. M_1_ = positive peak amplitude; M_2_ = positive peak width; M_3_ = negative peak amplitude; M_4_ = negative peak time. Red dotted line denotes latency of blink maximum or *T*_*0*_, corresponding to the moment when eyelids are fully closed. **(B)** Schematic illustration of data segmentation procedure. Blink epochs were centered at *T*_*0*_ (red solid lines), while control epochs were pseudo-random with respect to blink events. Number of epochs was identical between control and blink conditions for each subject. Black dotted line represents time zero for each condition. **(C)** Statistical contrasts were evaluated based on data in the intervals pre and post time zero for each condition (i.e., *Bpost* vs. *Bpre* for the blink condition, and *Cpost* vs. *Cpre* for the control condition).

To facilitate behavioral evaluation of blink instances across the group, quantitative features delineating different aspects of blink morphology were measured in the vEOG recording (Bonfiglio et al., 2014). Features were evaluated for each individual after averaging together all identified blink instances (Figure 1A). Since raw blink amplitudes vary across participants, the averaged blink trace for each individual was normalized relative to its own maximum amplitude prior to deriving the grand average waveform across participants (Figure 1A). This helped to minimize biasing of the grand averaged waveform due to any large signals at the subject level. Group-level statistics were performed to examine whether blink features were consistent across individuals using a split-half approach (Raz et al., 2014; Luking et al., 2017). Specifically, participants were randomly divided into two sub-groups of equal size, the values sorted, and Pearson correlation coefficient was calculated for each measure. This procedure was repeated 1,000 times, and the mean correlation coefficient was computed. The blink morphology was determined to be consistent across individuals if all extracted features showed mean correlation of greater than 0.8 over 1,000 repetitions.

### Data preprocessing

All subsequent analyses were performed on MEG data using a combination of SPM8 (Litvak et al., 2011) and EEGLAB (Delorme and Makeig, 2004). Data were band-pass filtered to 0.5–45 Hz, and independent component analysis (ICA) was performed using the runica algorithm (Makeig et al., 1996). Independent components corresponding to artifact (e.g., blinks, saccades, cardiac activity, muscle contractions, breathing) were removed.

To control for inherent brain activations that are not time-locked to blinks, we introduced an analytical control condition with pseudorandom timing with respect to blinks. Specifically, data were segmented in 3-s epochs according to two conditions (Figure 1B): a *blink* condition centered on the latency of blink maximum (*T*_0_) corresponding to complete eye closure, and a *control* condition segmented in non-overlapping consecutive epochs that were pseudo-random in timing with respect to blinks. For each individual, the control epochs were extracted near the middle of the 10-min dataset, and were matched with the blink epochs for number of trials. Statistical contrasts were evaluated between the intervals *pre-* and *post-* time zero for each condition (Figure 1C).

### Effectiveness of artifact removal procedure

To assess the effectiveness of the ICA-based artifact removal procedure, qualitative, and quantitative analyses were performed to evaluate the changes in spatial topography and regional signal power before and after artifact rejection. For each subject, the trial-averaged event-related fields (ERFs) time-locked to blinks were visually inspected pre- and post-artifact rejection to ensure removal of large-amplitude ocular artifact. In addition, the spatial topography for each individual was also evaluated at latencies corresponding to maximal blink amplitude (0 ms) and the pre-blink baseline (selected to be −1,000 ms to maintain separation from potential blink-related saccadic eye movements). Particular emphasis was placed on ensuring that frontally concentrated signals associated with blinking were no longer present following ICA artifact removal. Regional ERFs were also evaluated to examine the effect of artifact removal (Supplementary Materials).

In addition to qualitative assessments, quantitative analysis was also undertaken to measure the signal power at the blink latency and pre-blink baseline before and after artifact removal. For each subject, MEG sensors were first divided into subgroups based on their location, comprising the frontal, central, parietal, temporal, and occipital regions. For each region, the following ratio was derived to quantify the additional contribution of blink-related signals relative to that of baseline:

$$\begin{array}{l} ratio=\frac{\sum_{i=1}^{n}{\left(y_{i}-\bar{y}\right)}^{2}}{\sum_{i=1}^{n}{\left(x_{i}-\bar{x}\right)}^{2}} \end{array}$$

where *n* is the number of channels in each regional subgroup, and y_*i*_ and x_*i*_ are the ERF signals at 0 and –1,000 ms latencies, respectively. This provides a measure of the blink-to-baseline signal power while correcting for channel mean drifts. Thus, power ratios above unity would reflect greater signal at the blink latency compared to pre-blink baseline, while power ratios less than unity would reflect signal reduction relative to baseline. Statistical analysis was performed using paired *t*-test to compare the power ratios before and after ICA artifact removal in each region, with Bonferroni correction for multiple comparisons.

Since signals of ocular origin (such as blinks and saccades) have characteristic spatial patterns due to volume conduction of the ocular currents, further quantitative analyses were also conducted using one-way repeated-measures analysis of variance (ANOVA) to examine whether the changes in regional power ratios were consistent with this pattern (Supplementary Materials). Separate tests were undertaken before and after artifact removal.

### Sensor space

Sensor-space analysis employed global field power (GFP) to simultaneously capture information across all channels and account for differences in head position among individuals (Brunet et al., 2011; Mitchell and Cusack, 2011). GFP quantifies the spatial variance across all sensors at each time point (Skrandies, 1990), providing a measure of the signal power changes across all channels after correcting for channel mean drifts. GFP was derived using trial-averaged data in the delta band (0.5–4 Hz) for both *blink* and *control* conditions. To evaluate changes in GFP before and after time zero for each condition, mean GFP amplitudes were calculated over 200 ms windows in the *pre* and *post* intervals, and compared using paired *t*-test. The *post* window was chosen to surround the maximum GFP peak in the *blink* condition (150–350 ms), while the pre window was selected as −1,300 to −1,100 ms to represent baseline activity. Identical windows were used for both *blink* and *control* conditions.

To evaluate blink-related changes in sensor-level spectral power, time-frequency analysis was performed using continuous wavelet transform (CWT) with the Morlet function and six cycles. CWT was carried out for each channel and trial, and the log power was obtained as the logarithm of the squared absolute values of the coefficients within the frequency range of interest (defined to be 0.5–6 Hz to concentrate on the delta band). Baseline correction was performed by subtracting from each trial the mean log power in the baseline window, chosen to be −1,500 to −500 ms relative to *T*_*0*_ (Bonfiglio et al., 2013). Statistical significance was evaluated using a bootstrapping approach by permuting the trial-averaged wavelet power in the *pre* and *post* intervals in each frequency band across subjects.

### Source space

Following standard forward modeling in SPM8, we conducted source analysis to determine the neural generators of blink-related brain activity. Since this is the first MEG study of BROs, we chose the classical minimum norm estimates approach as this method does not require many prior assumptions about source characteristics (Dale and Sereno, 1993; Hauk, 2004). Group constraints were applied during inversion to ensure source reliability across subjects (Litvak and Friston, 2008; Litvak et al., 2011). Source reconstruction was based on raw trial-averaged data from all frequencies (0.5–45 Hz), using the entire 1,500 ms time window for each of the *pre* and *post* intervals. Source estimates were averaged over a predefined contrast time interval, projected to a three-dimensional source space, and smoothed using a Gaussian kernel of 8 mm full-width at half-maximum to create images of source activity for each subject. Statistical analyses were performed using a general linear model (GLM) for both *blink* and *control* conditions (Friston et al., 1994). T-contrast intervals were chosen to compare the peak post-blink activity (defined as the 200 ms window spanning the GFP peak) with that of pre-blink (defined as −1,300 to −1,100 ms). Contrast frequency was set to delta band.

Source-level time course activity was extracted by placing a virtual electrode in the centers of activation clusters within the bilateral precuneus regions. Voxel time courses were smoothed over a spherical volume of interest (VOI) with 5 mm radius, filtered to delta frequency, and averaged over trials. Statistical significance was evaluated using a bootstrapping approach with paired *t*-test: The *t*-statistic was calculated between the trial-averaged data for *blink* and *control* conditions at each time point in the epoch, and the size of the maximum suprathreshold cluster was extracted. The data were then permuted over all subjects and conditions, and randomly distributed into two groups. *T*-tests were repeated at each time point, and maximal suprathreshold clusters calculated. This process was repeated 5,000 times for each VOI time series to create a resampling distribution of suprathreshold cluster sizes. Statistical probabilities were derived by comparing the true cluster size outcomes with that of the resampling distribution.

## Results

### Behavioral

As expected, the total number of blinks varied among individuals (61 ± 34 blinks across group). The blink rate was 11.3 ± 6.9 blinks per minute, consistent with previous reports (Barbato et al., 2000). To evaluate blink behavior across subjects, morphological features were extracted from the unnormalized vEOG blink waveforms for each subject. Results are presented in Table 1. Group-level blink characteristics showed a positive peak of 43.70 ± 3.10 μV in amplitude, lasting for 0.329 ± 0.016 s. This is followed by a negative trough of −10.07 ± 1.13 μV in depth, occurring at a latency of 0.399 ± 0.017 s after *T*_*0*_. Consistency of blink features across individuals was evaluated using the split half approach by repeating the test 1,000 times following randomized group divisions, and the overall mean correlation coefficients were found to be extremely high for all measures (ρ = 0.89 for negative peak amplitude and ρ ≥ 0.95 for all other measures). These results indicate that the blink characteristics were highly consistent across subjects.

**Table 1:** Morphological features extracted from the unnormalized, trial-averaged vEOG waveform representing behavioral characteristics across subjects.

	**M_1_ Positive Peak Amplitude (μV)**	**M_2_ Positive Peak Width (s)**	**M_3_ Negative Peak Amplitude (μV)**	**M_4_ Negative Peak Time (s)**
Raw	43.70 ± 3.10	0.329 ± 0.016	−10.07 ± 1.13	0.399 ± 0.017
Correlation Coefficient (ρ)	0.95 ± 0.02	0.958 ± 0.029	0.89 ± 0.05	0.958 ± 0.025

*Raw = values extracted from unnormalized vEOG waveforms; reported as mean ± standard error across subjects. Correlation Coefficients = result of repeating split-half analysis 1,000 times after permuting the data; reported as mean ± standard deviation across repetitions*.

### Artifact removal

**Figure 2:**
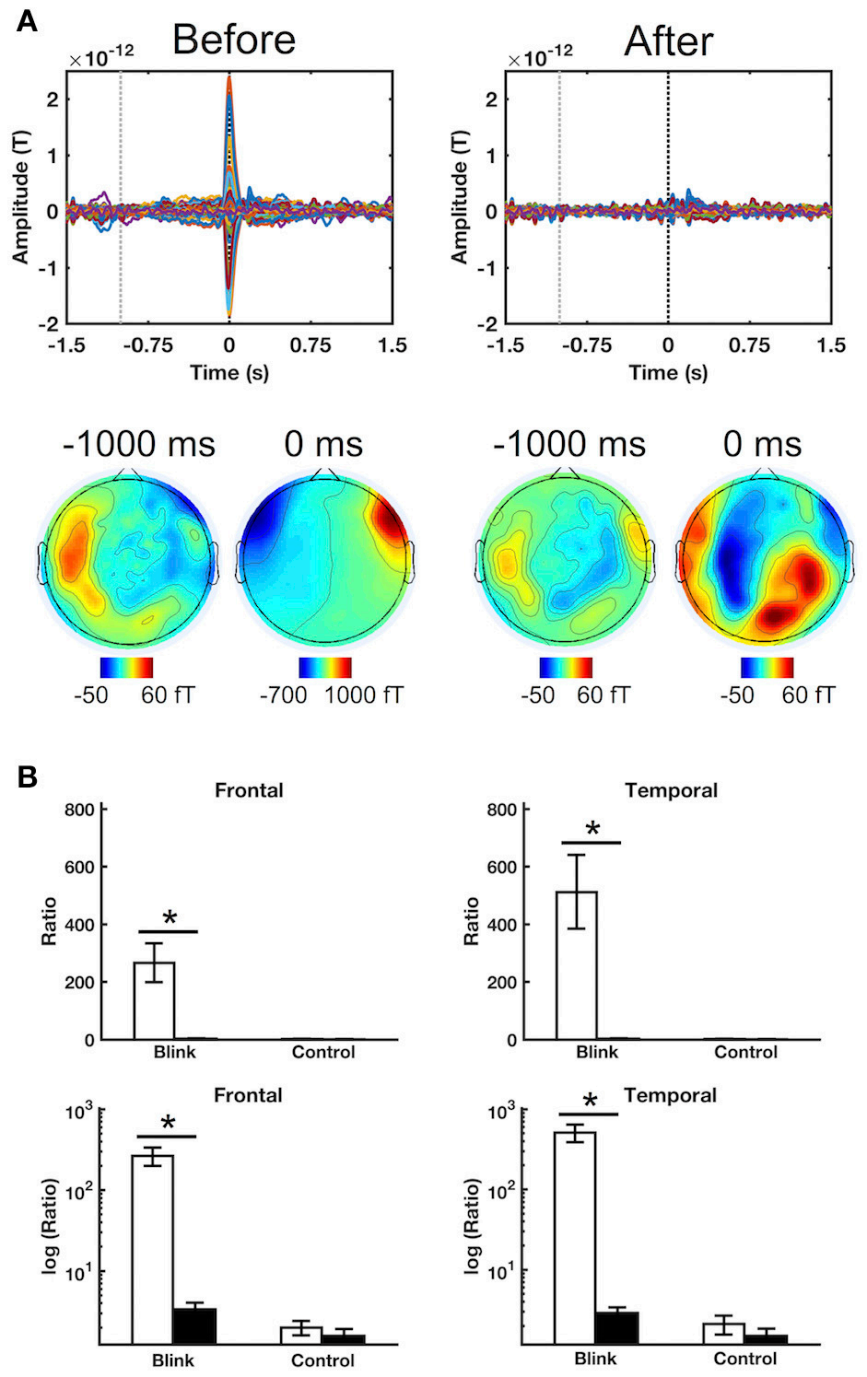
Sample results illustrating the efficacy of ICA artifact rejection. **(A)** ERFs for a representative subject before and after ICA artifact rejection. Top row shows time courses for all channels, while bottom row presents spatial topographies at the corresponding latencies. Black and gray dotted lines denote 0 ms (*T*_*0*_) and –1,000 ms latencies, respectively. **(B)** Regional blink-to-baseline power ratios before and after artifact rejection in the frontal and temporal channels where ocular artifact tends to be greatest. Top row shows raw power ratio values, in which the extreme discrepancy between the blink and control conditions led to obscuring of the control condition outcomes during display. The bottom row shows power ratios in logarithmic scale to improve display clarity. The ratios were computed for each subject and presented as mean ± SE across subjects. Statistical comparison was performed using raw, untransformed values. ^*^p < 0.005.

Qualitative evaluation of subject-level, all-channel ERFs before and after ICA artifact removal showed dramatic reduction in signal amplitude at the blink latency *T*_*0*_, as well as the disappearance of morphological features (e.g., spikes and boxes) generally considered to be indicative of blink and saccadic artifact (Figure 2A). These findings were also observed when sensor channels were separated into different spatial regions (Supplementary Figure 1). In addition, the spatial topography at the blink latency also underwent significant change, shifting from its concentration at the anterior eye regions to more central and posterior locations. These individual-level observations provide qualitative evidence regarding the effective removal of signal characteristics consistent with ocular sources.

In addition to qualitative assessments at the individual level, quantitative analysis was also undertaken to evaluate the impact of ICA artifact removal at the individual and group levels. Blink-to-baseline power ratio was used to evaluate the contribution of blink-related signals relative to that present in the pre-blink baseline for different spatial regions. Emphasis was placed on the frontal and temporal channels which are generally well-positioned to detect ocular signals. Results showed that the power ratio was significantly reduced in both of these regions for the *blink* condition following artifact removal (Figure 2B, *p* < 0.001). However, this was not observed in the *control* condition, for which the power ratio remained approximately unity (as *log*_10_ [ratio] ≈ 0). Similar results were also found in other regions (*p* < 0.005, Supplementary Figure 2). Cross-regional comparisons showed that, before artifact removal, the *blink* condition power ratio decreased from the temporal and frontal areas toward the posterior regions, consistent with the spatial propagation pattern of ocular currents (*p* < 0.005, Supplementary Materials). However, following artifact removal, no significant differences were found between regions, indicating that the spatial properties consistent with ocular artifact had been successfully removed from the data.

To ensure the complete removal of ocular signals following artifact rejection, source localization was also performed close to the blink latency, during the first 100 ms window immediately following blink maximum (Supplementary Figure 3). Results showed that, after artifact removal, activation clusters were located only in the posterior regions of the brain, and none were found in the anterior regions. This is consistent with the absence of activities associated with muscle contraction and eyelid movement, which have been shown to be localized to anterior brain regions (Bardouille et al., 2006).

### Sensor space

**Figure 3:**
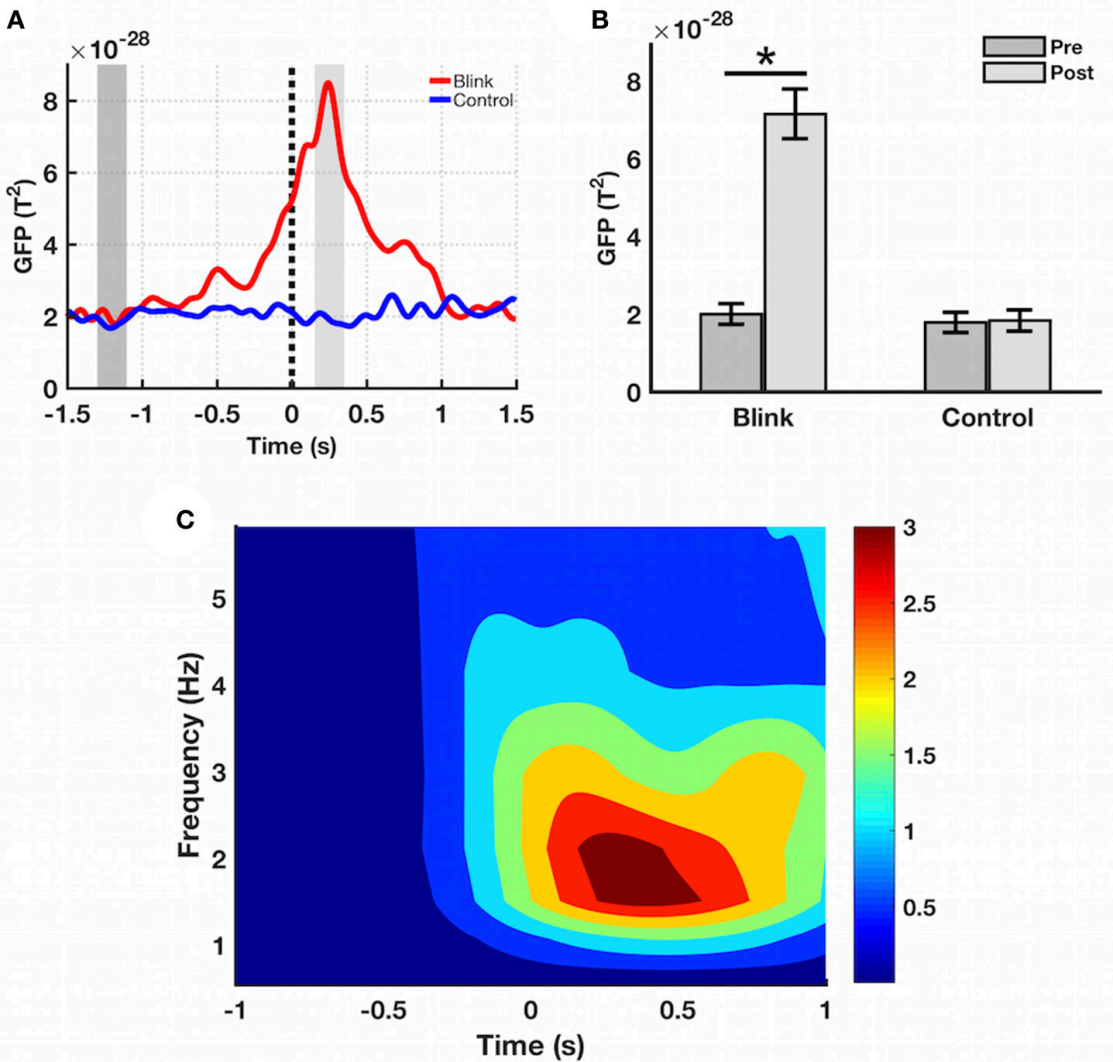
Sensor-level results. **(A)** Grand averaged GFP waveform in the delta band. GFP amplitude peaks at ~250 ms latency in the blink condition, not seen in control. Black dotted line denotes *T*_*0*_. Dark and light gray shaded regions represent windows of interest in the pre and post intervals, respectively. **(B)** Mean GFP over the windows of interest highlighted in part **(A)**, calculated for each subject and presented as mean ± SE over all subjects. ^*^p < 0.001. **(C)** Event-related spectral power in the blink condition, presented as mean over all channels. Delta band spectral power increases in the post-blink interval compared to pre-blink, peaking at ~250 ms latency (*p* < 0.05). Color bar represents power values.

Sensor-level time course activity of delta-band GFP showed increased post-blink power relative to pre-blink, peaking ~250 ms after *T*_*0*_ (Figure 3A). This was not observed in the *control* condition. To facilitate subsequent statistical analyses, two windows of interest were selected for the *post* and *pre* intervals based on the grand averaged GFP waveform, spanning the post-blink GFP peak (150–350 ms latency) and a corresponding pre-blink baseline (−1,300 to −1,100 ms latency), respectively. Using these windows, a paired *t*-test showed that the mean GFP power over the post-blink window was increased compared to pre-blink (*p* < 0.001), but these effects were absent in the *control* condition. Spectral analysis showed that event-related spectral power in the delta band was increased in the post-blink interval relative to pre-blink (*p* < 0.05), peaking at a similar latency compared to GFP.

### Source space

**Figure 4:**
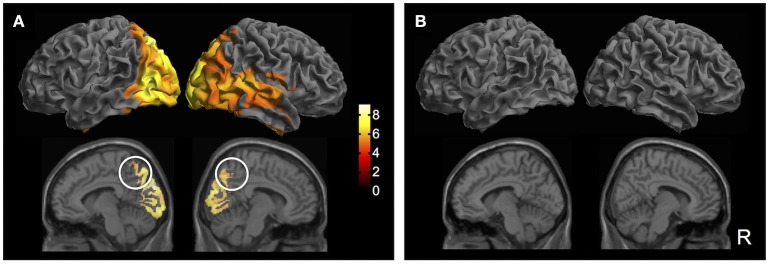
Source localization results (*p* < 0.05 FWE). **(A)** Bpost > Bpre contrast. Top row: Surface rendered maps showing activity across the bilateral dorsal and ventral visual streams spanning the occipital, posterior parietal, and inferior temporal regions. Right-lateralized activation of inferior frontal gyrus, posterior superior temporal gyrus, and anterior temporal lobe are also observed. Bottom row: Medial sagittal slices showing bilateral activation of the precuneus (white circles). Color bar shows *T*-statistic values. **(B)** Additional *T*-contrasts to examine blink-related reduction in activation (Bpre > Bpost) and activity inherent in the resting state (Cpost > Cpre; Cpre > Cpost). No suprathreshold activations were observed in any of the additional contrasts.

Building upon sensor-level results, the next step was to determine the neuroanatomical sources of the GFP peak activity. To this end, source-reconstructed time-frequency contrast maps were generated for the delta frequency band based on previously identified windows of interest. Results showed that, compared to pre-blink, post-blink activations were observed in the bilateral occipital, posterior parietal, and inferior temporal regions spanning the dorsal and ventral visual streams (*p* < 0.05 FWE, Bpost > Bpre contrast, Figure 4A). Additional blink-related activations were observed in the bilateral precuneus as well as right-lateralized regions within the inferior frontal gyrus, posterior superior temporal gyrus, and anterior temporal lobe (*p* < 0.05 FWE). To determine potential alternate patterns of activation, additional contrasts were introduced corresponding to blink-related reductions in brain activity (i.e., Bpre > Bpost), as well as any inherent brain activations in the passive fixation state that are not necessarily time-locked to blinks (i.e., Cpost > Cpre and vice versa). Importantly, none of the other contrasts resulted in any suprathreshold activations (Figure 4B). Furthermore, since the number of blinks varied across individuals, the total blink number was used as a covariate in the GLM to determine potential interactions between blink number and the experimental contrasts. No interactions were found.

**Figure 5:**
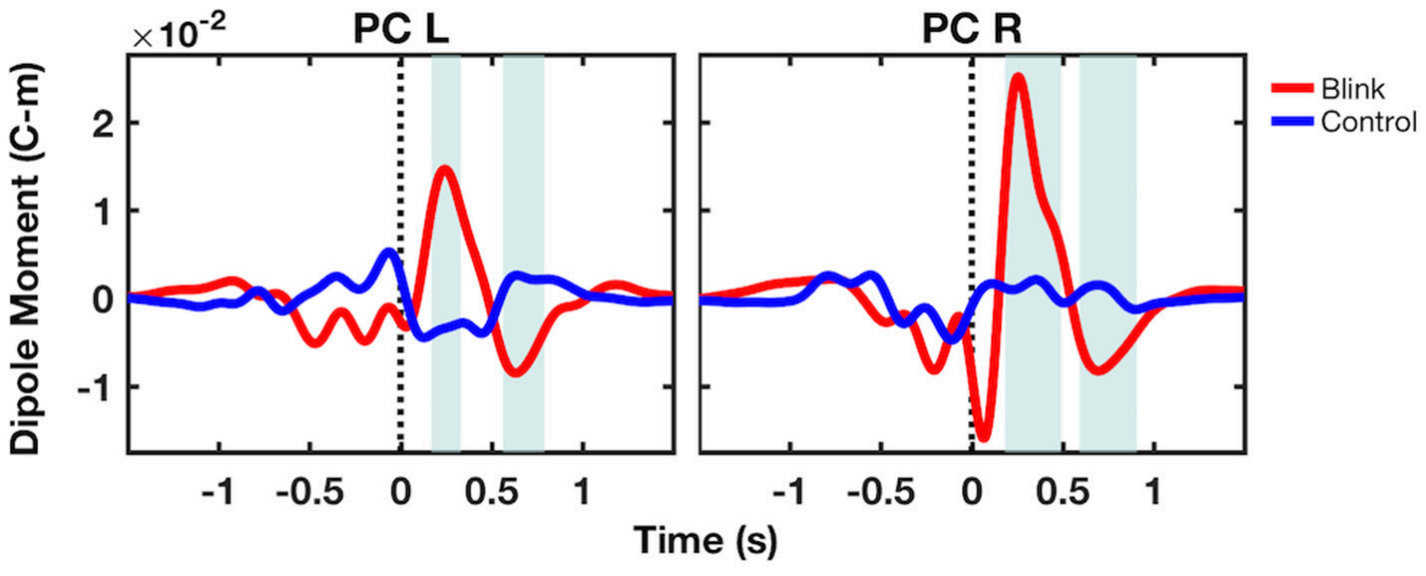
Grand-averaged virtual electrode time course activity from the bilateral precuneus. Shaded regions denote windows of significant difference between the blink and control conditions (*p* < 0.05). Black dotted line denotes *T*_*0*_. PC, precuneus; L, left; R, right.

To further examine source-level activity, time course data were extracted from virtual electrodes positioned at the center of two volumes of interest in the left and right precuneus (MNI coordinates [−8, −68, 49] and [7, −68, 39], respectively). Results showed that the bilateral precuneus exhibited large, positive deflections in the *blink* condition occurring ~250 ms post *T*_*0*_, followed by smaller negative deflections occurring at ~700 ms latency (Figure 5). These were not observed in the *control* condition. Permutation statistics showed that both the positive and negative deflections were significantly different from that of control (*p* < 0.05). Although the right precuneus also showed early negativity at ~100 ms latency, this was not significantly different compared to control.

## Discussion

### Main findings

As the first MEG study of BROs, we investigated the neurocognitive mechanisms of resting state delta-band (0.5–4 Hz) BROs associated with spontaneous blinking in healthy individuals. Utilizing simultaneous EOG to record ocular activity, we focused on neurological responses immediately preceding and following spontaneous blinks. Our main findings confirm our hypotheses that delta BRO activity measured using MEG would exhibit a large peak occurring in the 200–400 ms window post-blink (Hypothesis 1), the peak activity could be source-localized to the precuneus (Hypothesis 2), and that source-level activity within the precuneus would reflect the sensor-level characteristics (Hypothesis 3).

Sensor-level results showed increased GFP after spontaneous blinking compared to pre-blink baseline (Figures 3A,B), with peak power occurring ~250 ms post blink maximum. This is also reflected in the frequency domain, as sensor-level time-frequency analysis showed increased blink-related spectral power in the delta band relative to pre-blink baseline, peaking at a similar latency as the GFP activity (Figure 3C). Comparison between the *blink* and *control* conditions showed that no effects were present for the control condition, indicating that the observed GFP increase represented a *blink*-related brain response, independent of the ongoing resting state activity. These findings confirm our first hypothesis regarding sensor-level BRO activity, and are also consistent with previous reports of BROs using EEG (Bonfiglio et al., 2009, 2013).

Source localization results showed that spontaneous blinks led to increased activity in the bilateral precuneus, confirming our second hypothesis regarding the precuneal origins of delta BROs. Importantly, only the contrast corresponding to blink-related increases in activity produced suprathreshold activations (i.e., Bpost > Bpre); all other contrasts produced no suprathreshold activations, including those corresponding to the control condition (i.e., Bpre > Bpost; Cpost > Cpre; Cpre > Cpost). These results strengthen our assertion that the observed activations were due to spontaneous blinks rather than the ongoing resting state activity. Moreover, the number of blink trials was also incorporated as a covariate into our model to account for differences in blink rate across individuals. No significant effects were found due to trial number, providing further evidence that the observed effects were not due to any signal-to-noise ratio differences as a result of variability in trial number among individuals.

After extracting source-level time course activity using virtual electrodes, the grand-averaged dipole moment from the bilateral precuneus showed a large positivity at ~250 ms latency, followed by a negative deflection at ~700 ms (Figure 5). These effects were not observed in the *control* condition. Statistical analysis using bootstrapping showed that both observed effects were different compared to the *control* condition. The early negativity observed in the right precuneus dipole moment was not significantly different from control. In comparing the source level time course activity with that of GFP measured at the sensor level, it is apparent that both exhibit the peak at 250 ms. However, GFP is a measure of demeaned signal power across channels and therefore must be non-negative, while dipole moment can be either positive or negative. The comparison of GFP with dipole moment data thus indicates that the low positivity at ~700 ms latency in GFP may also be a reflection of the late negativity in dipole moment. These findings confirm our third hypothesis regarding the similarity between the source- and sensor-level characteristics.

### Study implications

To distinguish between blink-related brain activity and other ongoing activity inherent in the brain under passive fixation, this study employed a novel analytical control to account for the effect of time-locking with spontaneous blinks. In particular, while the *blink* condition was epoched such that trials were centered on spontaneous blink instances, a separate *control* condition was introduced for each subject that matched the corresponding *blink* condition in number of trials, but which was pseudo-random in timing with respect to blink instances. Because both control and blink epochs were derived from the same data and had equal number of trials, both were expected to reflect the same underlying neural activity and signal-to-noise ratio. However, only the *blink* condition was time-locked to spontaneous blink instances. The contrast between the two conditions should therefore provide information about the differential effects of spontaneous blinking relative to inherent brain activations. The fact that the observed effects were only present for the *blink* condition—and were absent in the *control* condition—lends further credence to our hypothesis that the BRO effects were specific to spontaneous blinks and were not part of ongoing brain activities in general.

The blink-related activation of bilateral precuneus regions observed in our study is consistent with previous reports of delta-band BROs using EEG (Bonfiglio et al., 2013, 2014). Activation of the precuneus is also consistent with fMRI studies of voluntary and spontaneous blinking (Bristow et al., 2005a; Nakano et al., 2013). The precuneus is a major hub in the brain, with one of the highest resting metabolic rates compared to other areas (Gusnard and Raichle, 2001). It has high anatomical and functional connectivity with other brain regions through extensive axonal networks (Hagmann et al., 2008; Greicius et al., 2009; Margulies et al., 2009; Zhang and Li, 2012). Moreover, it is also involved in high-level cognitive processes such as visuo-spatial imagery (Nagahama et al., 1999; Malouin et al., 2003; Wenderoth et al., 2005), episodic memory retrieval (Shallice et al., 1994; Gilboa et al., 2004; Lundstrom et al., 2005), and self-related processing (Kircher et al., 2000; Farrer and Frith, 2002; Kjaer et al., 2002).

Crucially, studies have shown that the precuneus is a major structural and functional core within the DMN (Fransson and Marrelec, 2008; Utevsky et al., 2014), the neuronal network that is highly activated in the absence of goal-directed tasks (including eyes-closed rest and eyes-open passive fixation), and represents the “default mode” of brain function (Raichle et al., 2001). The DMN encompasses the posterior cingulate cortex/precuneus, temporoparietal junction, medial prefrontal cortex, as well as inferior temporal and parahippocampal cortices (Gusnard and Raichle, 2001). Activity within these regions is suppressed during active task conditions, suggesting that the brain suspends or disengages ongoing, internally directed processes in order to facilitate the reallocation of neuronal resources to meet external attentional demands (Raichle et al., 2001). In fact, the prevailing hypothesis regarding the significance of this activity is that the precuneus and other DMN regions are continually engaged in both information gathering from the environment as well as representations of the self within this environment (Buckner et al., 2008; Andrews-Hanna, 2012). In other words, in the absence of external attentional demands, the brain's default state is to both monitor the environment as well as track and evaluate the past, present, and future states of the self within this context. The observed precuneal activation immediately following each blink in the current study is thus consistent with the brain's continually monitoring the environment with each new image that appears after the eyelids reopen (Bonfiglio et al., 2011, 2013).

Our findings are further supported by a previous MEG study examining voluntary blinking while individuals fixated on a cross or small pictorial image. Hari et al. demonstrated that activation of the medial parieto-occiptal sulcus was observed only when blinking occurred in light conditions, and was not found when blinking occurred in complete darkness (Hari et al., 1994). This is consistent with the environmental awareness aspects of blink-related precuneal activation, since visual input about the environmental must be a prerequisite to monitoring and awareness of such environment. It should be noted a previous EEG-based BRO study also reported activation of the posterior cingulate cortex in 3 of their 11 subjects (Bonfiglio et al., 2013), which is not seen in the current study. However, this is likely due to the difference in imaging modality between the two studies, as the low-density EEG system used in that study would have had limited spatial resolution compared to MEG.

The development of BROs as a potential brain function marker may also have important clinical implications, as the DMN—especially the precuneus region—has been shown to be critical to the brain's ability to sustain consciousness (Boly et al., 2013). For instance, functional connectivity between DMN regions—particularly between the precuneus and frontal areas—was negatively correlated with the level of clinical consciousness impairment in brain-injured patients (Vanhaudenhuyse et al., 2010). In addition, changes in functional connectivity between DMN regions have also been found to be correlated with both the level of functional impairment in patients with traumatic brain injury (Sharp et al., 2011) as well as the degree of recovery in these patients (Hillary et al., 2011). These findings help to highlight the clinical significance of DMN activity as a potential avenue for evaluating consciousness. However, the use of fMRI as the imaging modality in these studies severely limits accessibility at the bedside, emphasizing the need to create new tools for assessing this activity in a point-of-care setting. In light of this, the development of BROs as an electromagnetic/electrophysiological indicator of precuneus activity may provide an EEG-based assessment tool for consciousness (Bonfiglio et al., 2013, 2014), with improved capacity for bedside deployment. Additionally, the implication of spontaneous blinking in various neurological disorders such as Parkinson's disease and schizophrenia also presents another potential avenue for the application of BRO brain processes in clinical evaluations (Chan et al., 2010; Bologna et al., 2013).

The blink-related occipital activations in this study are consistent with previous fMRI reports of blinking, some of which associated these activations with the phenomenon of blink suppression (Bristow et al., 2005b; Berman et al., 2012). However, the use of MEG in the current study provides greater insight into the temporal dynamics of blink-related neural activity, enabling the examination of brain activity immediately before and after a blink instance—which is not possible using fMRI. Blink suppression has been shown to begin ~150 ms prior to blink onset and recover by 200 ms after blink onset (Volkmann, 1986). Using zero voltage as the baseline for extracting blink onset points in the current study, the interval of blink suppression thus ends ~50 ms after *T*_*0*_. This is much earlier than the peak latency of 250 ms relative to *T*_*0*_ observed in both GFP and virtual electrode time course data in this study, thereby rendering blink suppression an unlikely explanation for the observed occipital activations. Instead, it is more likely that the bilateral activations across the occipital, posterior parietal, as well as inferior temporal regions represent activity within the ventral and dorsal streams of visual processing as the brain examines each new image following a blink (Hebart and Hesselmann, 2012).

The blink-related occipital activations in this study are also consistent with previous work using MEG (Hari et al., 1994; Bardouille et al., 2006) and EEG (Heuser-Link et al., 1992). These studies examined the spatiotemporal characteristics of ocular currents due to voluntary blinking in both light and dark conditions, and demonstrated that occipital activations occurred only in the light condition—consistent with visual processing (Heuser-Link et al., 1992; Hari et al., 1994; Bardouille et al., 2006). In contrast, source activity associated with muscle contraction and ocular currents were localized to the frontally concentrated eye regions, and were observed in both light and dark conditions (Heuser-Link et al., 1992; Bardouille et al., 2006). Since the current study examined spontaneous blinking under light conditions, these findings lend further credence to our hypothesis that the blink-related occipital activations observed in this study were associated with visual processing following eye opening.

To examine the variability of sensor-level GFP waveform morphology across individuals, we also undertook further analyses to compute 95% confidence intervals (CIs) for each time point in the grand-averaged GFP waveform (Supplementary Materials). Results showed relatively narrow CIs for both blink and control conditions throughout the epoch, without any overlaps between the *blink* and *control* condition CIs in the 0–500 ms window post-blink. These findings suggest that the GFP waveform morphology was relatively consistent across individuals, and help to strengthen our observations regarding the GFP peak morphology. In addition, we also undertook analyses to examine individual sensor ERF waveforms for comparison with the virtual electrode time course data (Supplementary Materials). Both ERF and virtual electrode activities exhibited multiple peaks of positive and negative polarities occurring at different latencies within the first 750 ms window post-blink. Although there is insufficient evidence in this study to propose neurophysiological mechanisms involved in blink processing, we speculate that blink-related neural responses involve a combination of visual and environmental monitoring processes, with possible involvement of episodic memory. The occipital activations point to potential sensory processing of visual input associated with each new blink, while the precuneal activations may represent further information processing in the context of environmental monitoring. Additionally, given the known involvement of the precuneus in episodic memory processes (Gilboa et al., 2004), this may also involve comparison of the new information content with that in prior experiences. Nonetheless, further studies are needed to validate these speculative hypotheses.

Additional right-lateralized activations were observed in the right inferior frontal gyrus as well as the superior, middle, and inferior temporal gyri. We speculate that the right-lateralized inferior frontal and superior temporal activations may be related to the ventral attention network in the context of post-blink detection of sensory stimuli that may have potential behavioral relevance (Corbetta and Shulman, 2002). On the other hand, activation of the right temporal lobe has been associated with processing of pictorial inputs (Snowden et al., 2004), social concepts (Zahn et al., 2007), person knowledge (Bethmann et al., 2012), or emotional processing (Reiman et al., 1997). Further studies are needed to elucidate the potential roles of both the ventral attention network and the other right temporal lobe activations within the context of spontaneous blinking.

### Caveats

As the first MEG study of BROs, the present work utilized a distributed source modeling approach with minimum norm estimates for localizing cortical sources of blink-related activity. Although this approach requires few prior assumptions regarding source characteristics, it does have inherent limitations in biasing toward sources closer to the cortical surface (Hauk, 2004). Further studies are needed to validate the source space results using alternate source reconstruction approaches such as spatial filtering with beamformer (Hillebrand et al., 2005). In addition, the potential role of the ventral attention network in spontaneous blinking should be examined through network analysis.

## Conclusions

Our study investigated the neurocognitive mechanisms of delta-band BROs using MEG. Results showed that spontaneous blinks activate the bilateral precuneus associated with environmental monitoring and self-awareness. BROs were time-locked to spontaneous blinks and were not part of the inherent brain activity associated with passive fixation, and responses were consistent at both the sensor and source levels. As the first MEG study of BROs, these findings provide neuroimaging support for the importance of examining blink-related brain activity.

## Author contributions

Data collection: CL, and SGH; Analysis and interpretation: CL, and SGH; Study design: CL; Study funding: CL, TC, and RD; Drafting manuscript: CL. All authors commented on the manuscript and approved the final draft. This publication is the original work of the authors and RD will serve as guarantor of its contents.

### Conflict of interest statement

The authors declare that the research was conducted in the absence of any commercial or financial relationships that could be construed as a potential conflict of interest.
