# Supplementary material for: Spontaneous Blinks Activate the Precuneus: Characterizing Blink-Related Oscillations Using Magnetoencephalography
Source: Front Hum Neurosci. 2017 Oct 11;11:489. doi: 10.3389/fnhum.2017.00489 (PMC5649156; doi:10.3389/fnhum.2017.00489)
Supplement: Supplementary file 1 [file DataSheet1.pdf]

frontiers

Supplementary Material

Spontaneous Blinks Activate the Precuneus: Characterizing Blink-Related Oscillations Using Magnetoencephalography

Careesa C. Liu, Sujoy Ghosh Hajra, Teresa Cheung, Xiaowei Song, Ryan C.N. D'Arcy*

*** Correspondence:** Ryan C.N. D'Arcy: rdarcy@sfu.ca

1 Qualitative Effects of Artifact Removal: Regional ERFs

Sample results demonstrating the regional impact of artifact removal are shown in Supplementary Figure 1. Before data cleaning, prominent artifact from both blink and saccadic sources can be observed in the temporal and frontal channels, with gradual reduction in artifact amplitude toward posterior areas. This is consistent with the expected signal propagation pattern of ocular artifact. After ICA, no apparent traces of blink and saccadic artifact remain in any of the regional ERFs.

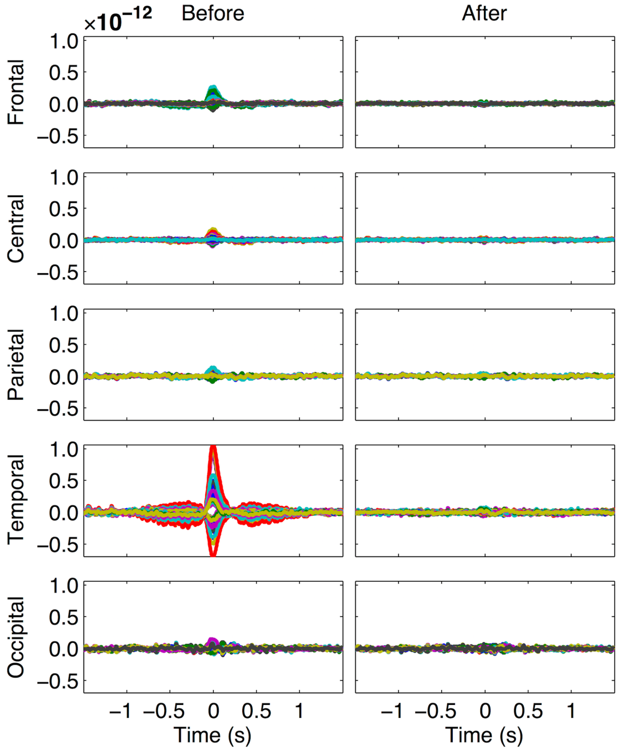

Supplementary Figure 1. Data from a representative subject showing trial-averaged ERF in the blink condition, before and after artifact removal. Sensor channels in different regions are displayed separately to illustrate the effect of ICA at different sensor locations.

2 Quantitative Effects of Artifact Removal: Before vs. After Comparison

Regional blink-to-baseline power ratio was measured in the trial-averaged ERF before and after artifact removal (Supplementary Figure 2). In the blink condition, there was significant reduction in power ratio in all regions ($p<0.005$). This decrease was greatest in the frontal and temporal channels where ocular artifact was expected to be highest (98% and 99% reduction, respectively). Channels in the central and parietal regions also exhibited high levels of power reduction following artifact removal (96% and 92%, respectively), while the occipital channels showed the lowest power decrease (76%). These results are consistent with the reduction in ocular signal amplitude as the field propagates in the anterior-posterior direction, leading to greater impact of ICA artifact removal in the anterior compared to posterior regions. In contrast, the power ratios were approximately 1 (as $log_{10}(\text{ratio}) \approx 0$) for all regions in the control condition, both before and after artifact removal. Since the control condition was pseudo-random in timing with respect to blink and was therefore not expected to reflect much blink-related activity in the trial-averaged ERF, the signal amplitudes at both 0ms and -1000ms latencies were not expected to differ in this condition. Given that ocular activity generally represents the largest source of signal artifact, the application of ICA artifact removal was therefore not expected to have as much impact on the control condition compared to blink.

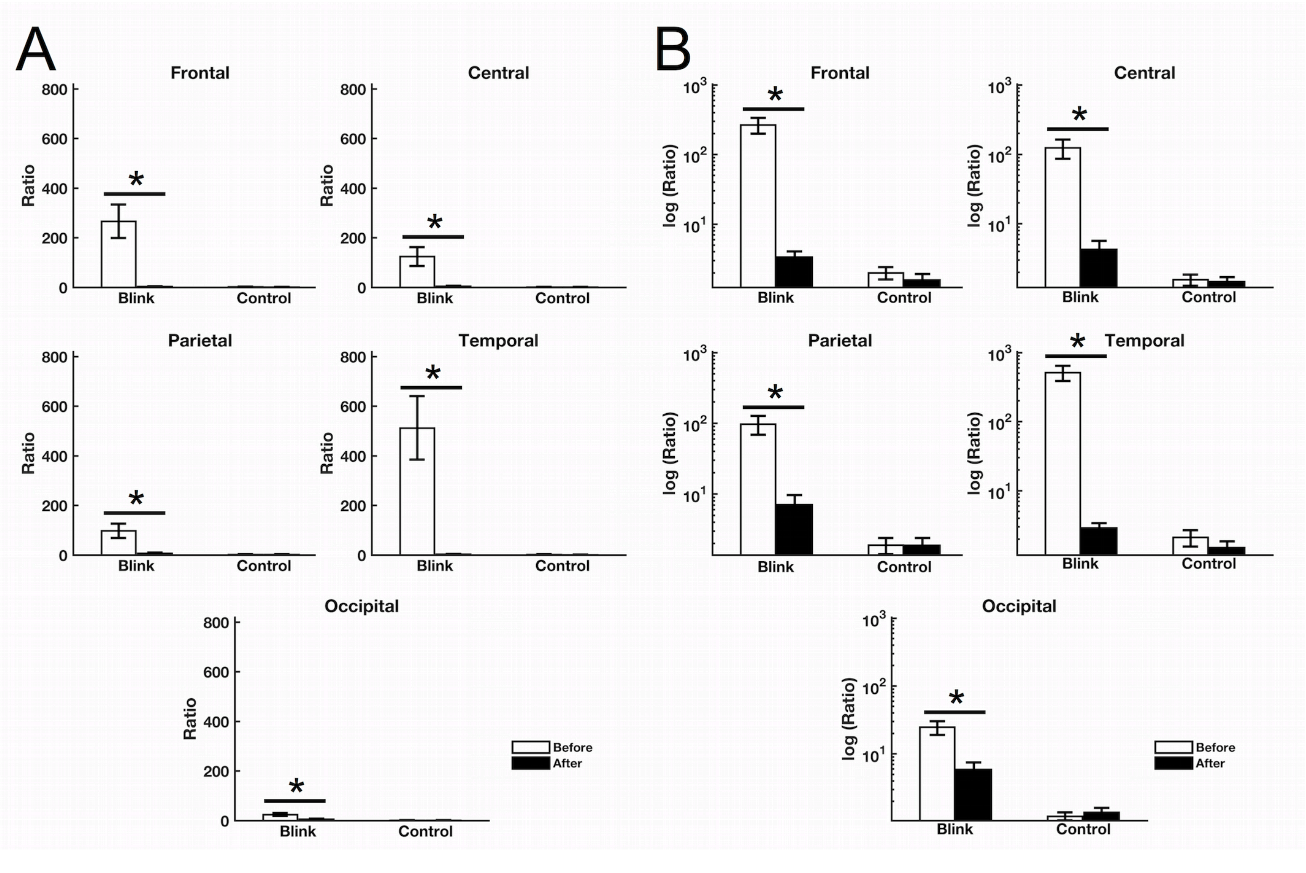

Supplementary Figure 2. Regional blink-to-baseline power ratio before and after artifact removal, calculated for each subject and presented as mean ± SE across subjects. A) Raw power ratios. Blink condition showed dramatic reduction following artifact removal, while the control condition outcomes were obscured during display due to the extreme discrepancy in power ratio values

between the two conditions. B) To improve display clarity, power ratios are also presented in logarithmic scale. No reductions in power ratio are observed in the control condition. *p<0.005 paired t-test.

3 Quantitative Effects of Artifact Removal: Spatial Distribution

To further evaluate the effect of artifact removal at the group level, additional statistical analyses were performed using one-way repeated-measures analysis of variance (ANOVA) in SPSS Statistics 23 (IBM), with 'region' as a within-subject factor. Results showed that, prior to artifact rejection, there was a significant region effect on the blink condition power ratios ($F_{1.225,42.863}$ = 12.934, p<0.0001). Pair-wise comparisons with Bonferroni correction showed that power ratios from all regions were different from those of all other regions except for the central and parietal locations (p<0.005). The overall pattern of progression in the power ratio was found to be as follows:

$$\textit{Temporal} \overset{*}{>} \textit{Frontal} \overset{*}{>} (\textit{Central} \approx \textit{Parietal}) \overset{*}{>} \textit{Occipital}, \text{ where * indicates } p<0.05$$

Since both the lateral frontal and anterior temporal channels are generally well-positioned to detect ocular signals, this reaffirms the expected pattern of spatial propagation for ocular currents.

Contrary to the pre-ICA results, the region effect on the blink condition power ratios completely disappeared following artifact rejection ($F_{1.816,63.572}$ = 1.531, p=0.225). This strongly suggests that the spatial properties associated with the ocular signal were no longer present, indicating that ocular artifacts had been successfully removed through the ICA artifact rejection process.

It is important to note that the same statistical tests were also performed for the control condition, both before and after artifact rejection. No significance was found in either instance ($F_{2.822,98.768}$ = 1.081, p=0.359 before rejection; $F_{2.106,73.718}$ = 0.418, p=0.670 after rejection). Together, these results demonstrate the successful removal of ocular artifact through ICA.

4 Source Localization at Blink Latency

To ensure the complete removal of ocular artifact, we also performed source localization analysis using a time window immediately following blink maximum (from 0 to 100ms latency). All other parameters were identical to the analysis in the manuscript. Results are shown in Supplementary Figure 3 below. Several small activation clusters were observed over the posterior occipital and temporal regions, and none were found in the anterior regions. In contrast, other EEG and MEG studies of blink-related brain activations reported that activities associated with ocular artifact (i.e. muscle contractions and eye movement) were source localized to the anterior eye regions (Bardouille, Picton, & Ross, 2006; Heuser-Link, Dirlich, Berg, Vogl, & Scherg, 1992). Since no anterior activations were found in our study following artifact removal, this suggests that ocular artifact-related activity was not present in the data.

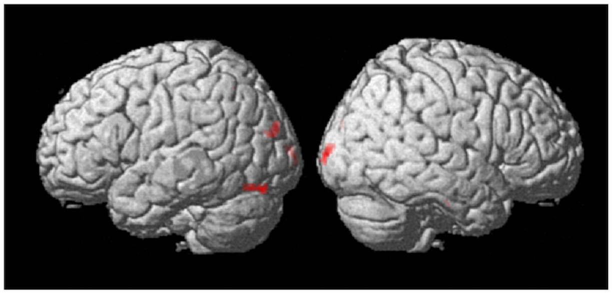

Supplementary Figure 3. Source localization results for the 0 to 100ms window following artifact removal for Bpost > Bpre contrast ($p<0.05$ FWE). No activations were observed in the anterior regions.

5 Consistency of GFP Results Across Individuals

To evaluate the consistency of GFP results across individuals, 95% confidence intervals were calculated for each time point in the GFP waveform (Supplementary Figure 4). Results showed that confidence intervals were relatively narrow, without any overlaps between the blink and control confidence intervals in the 0-500ms window post-blink. These results suggest that waveform morphology was relatively consistent across subjects.

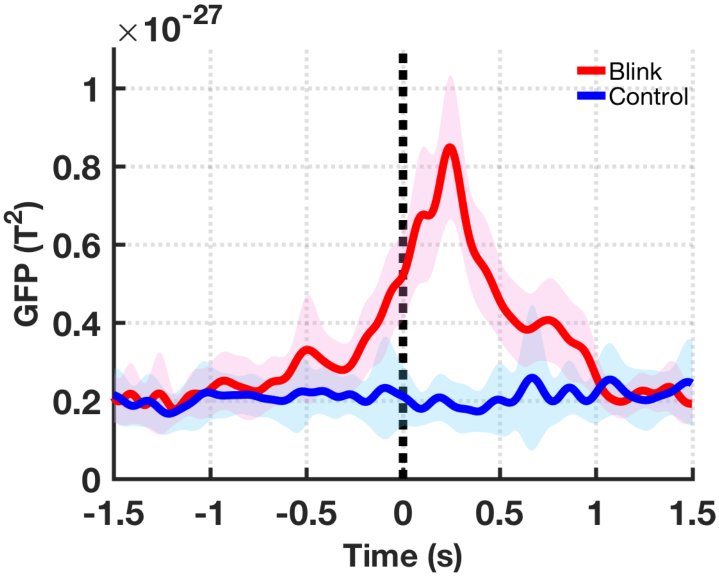

Supplementary Figure 4. GFP results showing grand-averaged waveforms in solid lines. Shaded regions denote the corresponding 95% confidence intervals across subjects.

6 Individual ERF

Although sensor-level analysis in our study focused on GFP which is blind to signal polarity and showed a single peak maximal at 250ms, the source-level time course activity within the precuneus did exhibit multiple peaks of both positive and negative polarities within the first 750ms post-blink. We hypothesized that these peaks may be reflected in the smaller positivities observed in the GFP surrounding the main peak. To further explore this issue, we also examined the individual

ERF waveforms in sensor space (Supplementary Figure 5). Results showed the presence of multiple peaks of both positive and negative polarities in the blink condition ERF waveforms within the first 500ms post-blink, which were absent in the control condition. These observations are similar to those from source-level precuneus time courses, and help to support our hypothesis that the GFP waveforms reflect activity from multiple sub-peaks.

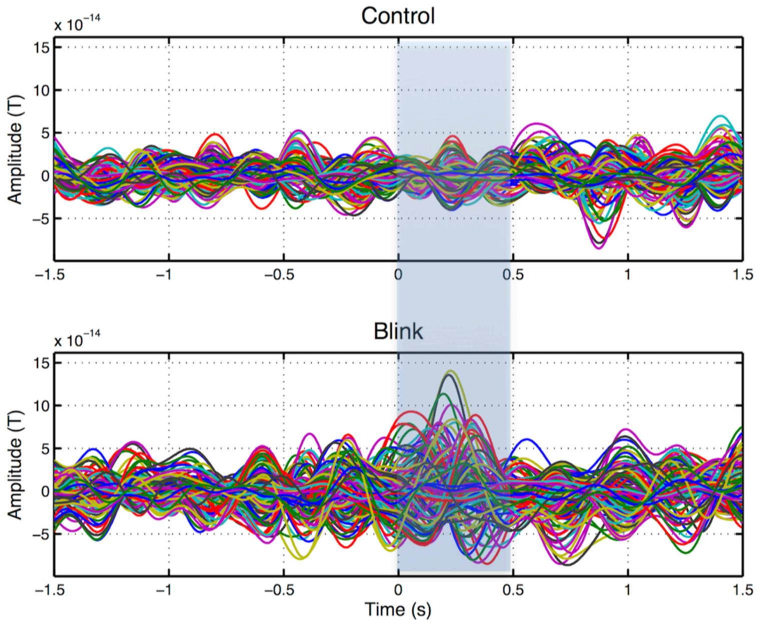

Supplementary Figure 5. Sample ERF waveforms for a representative subject showing both control and blink conditions. Shaded region denotes the 0-500ms window in which multiple peaks are observed in the blink condition.

7 MRI Co-Registration Results

Standard co-registration using SPM8 was performed in this study. However, because subject-specific MRIs could not be obtained for this study, an optimization approach was also implemented to select the best-fit surrogate structural MRI from a database of 30 adult structural MRI datasets ($1mm^3$ voxel resolution) (Peatfield, Wen, Talpalaru, & Cheung, 2017). Briefly, coordinates between the polhemus-digitized head shape and the MEG data were first co-registered using the three fiducial coils. The scalp mesh points for each candidate MRI were then computed using SPM8, and a surface-matching procedure was conducted by calculating the nearest neighbour points between the structural MRI mesh and the polhemus digitization points. The candidate MRI with the smallest mean nearest neighbour distance was selected as the surrogate structural MRI. This method has previously been shown to achieve comparable co-registration results relative to subject-specific MRIs in MEG source modeling for auditory evoked fields (Peatfield et al., 2017). In the current study, the polhemus points for each subject were superimposed on top of the surrogate MRI for that subject, and the results were visually inspected to ensure a close match between the structural MRI and the polhemus points (Supplementary Figure 6).

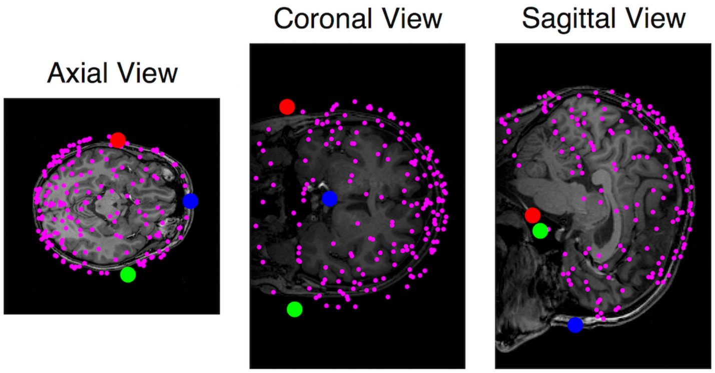

Supplementary Figure 6. Sample co-registration results for representative subject showing polhemus digitization points (pink) overlayed on top of the structural MRI. The fiducial coils are also shown (blue = nasion; red = left peri-auricular; green = right peri-auricular).

References

Bardouille, T., Picton, T. W., & Ross, B. (2006). Correlates of eye blinking as determined by synthetic aperture magnetometry. *Clinical Neurophysiology, 117*(5), 952-958. doi:http://dx.doi.org.proxy.lib.sfu.ca/10.1016/j.clinph.2006.01.021

Heuser-Link, M., Dirlich, G., Berg, P., Vogl, L., & Scherg, M. (1992). Eyeblinks evoke potentials in the occipital brain region. *Neuroscience Letters, 143*(1-2), 31-34.

Peatfield, N., Wen, A., Talpalaru, A., & Cheung, T. (2017). Testing the effectiveness of dummy MRIs in place of actual MRIs when doing co-registration with MEG. *Organization for Human Brain Mapping (OHBM),* Vancouver, Canada.
